# Case Report: An exceptional response to neoadjuvant radiotherapy and chemotherapy in undifferentiated pleomorphic sarcoma following checkpoint inhibitor use

**DOI:** 10.3389/fonc.2023.1198292

**Published:** 2023-06-23

**Authors:** Won Jin Jeon, Jin Hyun Moon, Bryan Pham, Bowon Joung, Laura Denham, Joel Brothers

**Affiliations:** ^1^ Department of Internal Medicine, Loma Linda University, Loma Linda, CA, United States; ^2^ Department of Anatomic and Clinical Pathology, Loma Linda University, Loma Linda, CA, United States; ^3^ Division of Medical Oncology and Hematology, Department of Internal Medicine, Loma Linda University, Loma Linda, CA, United States

**Keywords:** undifferentiated pleomorphic sarcoma (UPS), immune-checkpoint inhibitor (ICI), perioperative therapy, tumor microenvironment, neoadjuvant therapy

## Abstract

Undifferentiated pleomorphic sarcoma (UPS), a subtype of soft tissue sarcoma (STS), is an uncommon malignancy associated with a poor prognosis. As with other forms of sarcoma, surgical resection remains the only form of treatment with curative potential. The role of perioperative systemic therapy has not been definitively elucidated. Due to high recurrence rates and metastatic potential, management of UPS can pose a difficult task for clinicians. In cases of unresectable UPS due to anatomic limitations and in patients with comorbidities and poor performance status (PS), management options are limited. We describe a patient with UPS involving the chest wall with poor PS who achieved complete response (CR) following neoadjuvant chemotherapy and radiation in the setting of prior immune-checkpoint inhibitor (ICI) therapy.

## 1 Introduction

STS is a rare cancer, comprising less than 1% of all new cases of adult malignancies (1). According to data from the Surveillance, Epidemiology, and End Results (SEER) program, UPS comprises 17.1% of cases of STSs (2). The mainstay of treatment for UPS is surgical resection with adequate margins, with consideration given to perioperative RT and chemotherapy (3, 4). UPS is notorious for a high rate of recurrence with high metastatic potential, making the management of UPS difficult, and the prognosis is poor for patients with metastatic or surgically unresectable disease (4). Here, we present a patient with UPS involving the chest wall who received treatment with ICIs prior to the diagnosis of UPS. She had an excellent response to palliative-intent radiotherapy, enabling her to undergo neoadjuvant chemotherapy and potentially curative surgical resection.

## 2 Case description

**Figure 1:**
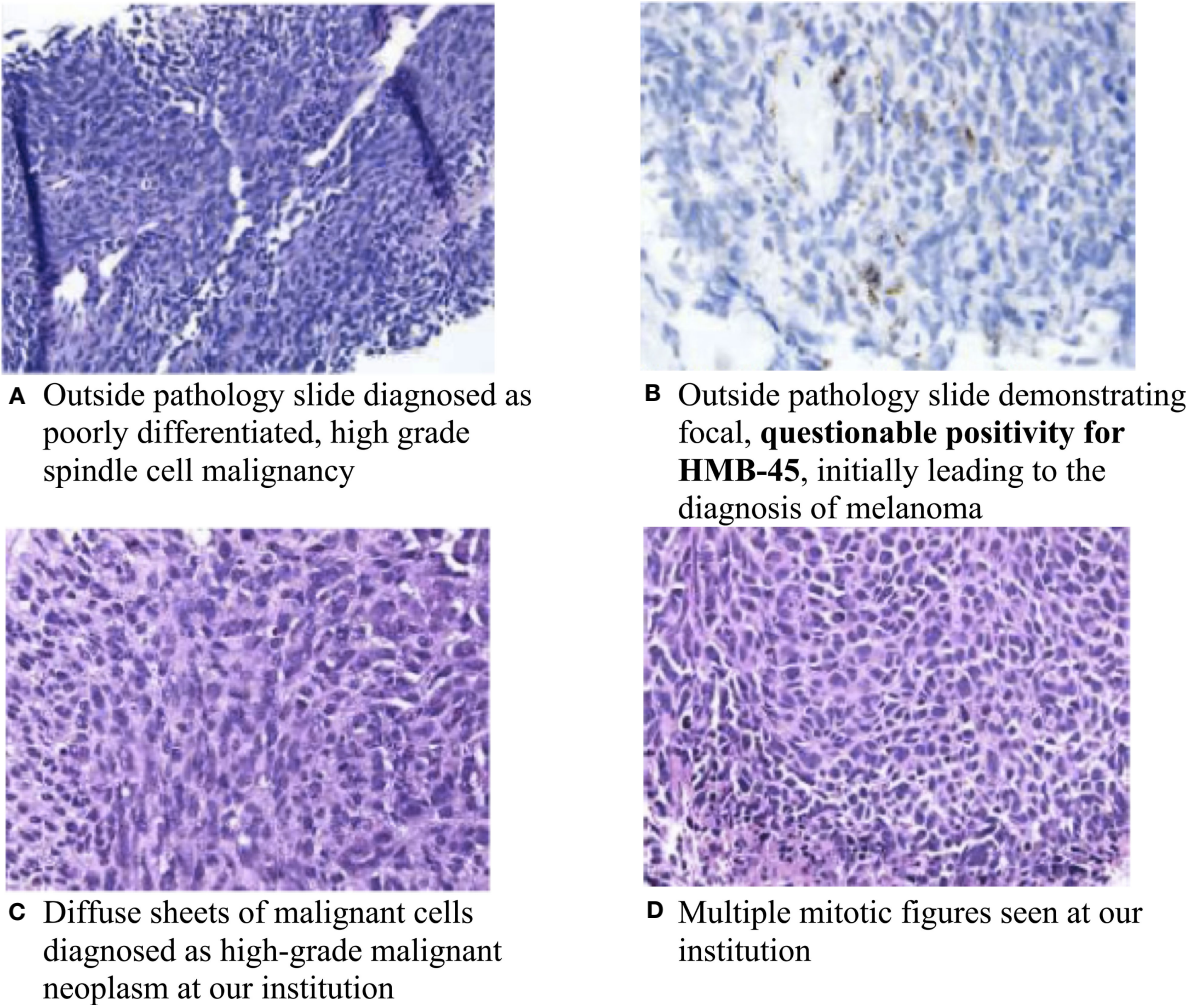
Pathology slides of the left chest wall mass. **(A)** Outside pathology slide diagnosed as poorly differentiated, high-grade spindle cell malignancy; **(B)** Outside pathology slide demonstrating focal, questionable positivity for HMB-45, initially leading to the diagnosis of melanoma; **(C)** Diffuse sheets of malignant cells diagnosed as high-grade malignant neoplasm at our institution; and **(D)** Multiple mitotic figures seen at our institution.

A 69-year-old female with no significant past medical history presented to an outside institution with a progressively enlarged left axillary lesion. A left upper extremity deep venous thrombosis (DVT) was discovered, and treatment with rivaroxaban was initiated. A biopsy of the axillary mass revealed poorly differentiated, high-grade spindle cell malignancy. Immunohistochemistry (IHC) was positive for vimentin and showed questionable focal HMB-45 positivity (**Figures 1A, B**). Based on the focal positive HMB-45 stain, the patient was given a presumptive diagnosis of cutaneous melanoma, and she received a single cycle of ipilimumab/nivolumab.

**Figure 2:**
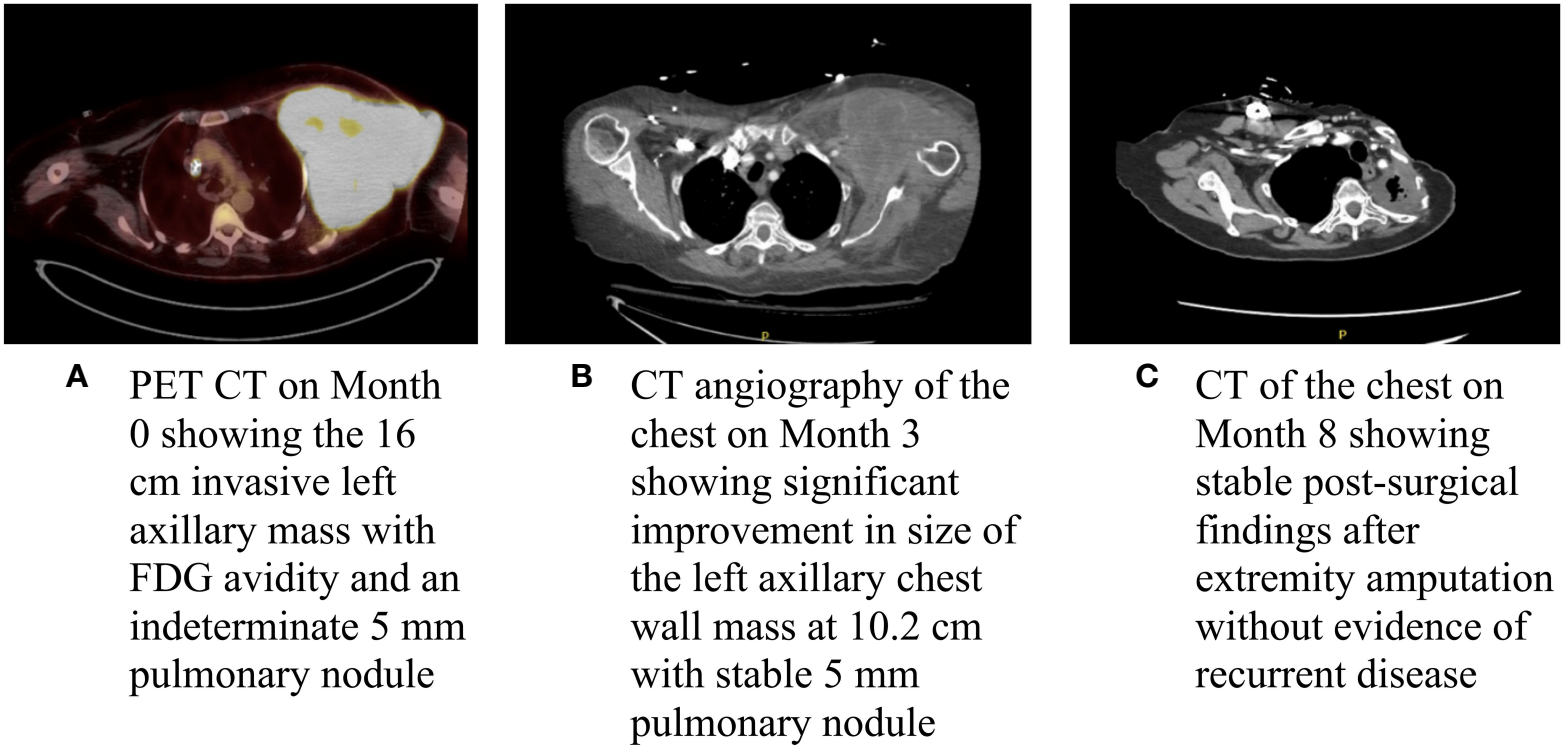
Baseline and interval imaging of the UPS of the chest wall. **(A)** PET CT on Month 0 showing the 16 cm invasive left axillary mass with FDG avidity and an indeterminate 5 mm pulmonary module; **(B)** CT angiography of the chest on Month 3 showing significant improvement in size of the left axillary chest wall mass at 10.2 cm with a stable 5 mm pulmonary nodule; and **(C)** CT of the chest on Month 8 showing stable post-surgical findings after extremity amputation without evidence of recurrent disease.

About a month later, the patient presented to the emergency department (ED) at our institution with acute bleeding and foul-smelling drainage from the left axillary mass, with associated subjective fever. The patient was admitted for acute blood loss, anemia, hypercalcemia, and sepsis. Computed tomography (CT) of the chest showed a necrotic, fungating 17 × 15 × 15 cm left axillary mass with invasion into the left chest wall. CT of the abdomen and pelvis and magnetic resonance imaging (MRI) of the brain were negative for metastatic disease. Positron emission tomography-CT (PET-CT) demonstrated a 16 cm invasive left axillary mass with FDG avidity and an indeterminate 5 mm pulmonary nodule below PET resolution (**Figure 2A**). A repeat biopsy was performed and compared to the outside biopsy specimen by our institution’s pathologists (**Figures 1C, D**)*. I*HC stains for melanoma (HMB-45, S-100, melanoma cocktail, and SOX10), carcinoma, and markers of neural, muscular, and vascular differentiation were all negative, favoring a diagnosis of UPS (**Table 1**). Tumor-infiltrating lymphocytes (TILs) were estimated to be 1%–3% in the post-immunotherapy biopsy, and there was a noticeable increase in single-cell necrosis. TILs were not able to be estimated from the pre-treatment biopsy. After evaluation by surgical oncology and medical oncology, the patient was deemed a poor surgical and inpatient chemotherapy candidate due to poor PS (Eastern Cooperative Oncology Group (ECOG) of 4). Radiation oncology administered a single fraction of palliative RT (800 cGy) in the hospital. The patient was discharged with a plan for outpatient medical oncology follow-up.

**Table 1:** Immunohistochemical (IHC) results.

Antibody/ Stain	Result
**Vimentin**	**Positive**
**HMB-45**	**Negative**
**S-100**	**Negative**
**Melanoma cocktail**	**Negative**
SOX-10	Negative
Pancytokeratin	Negative
p63	Negative
CK CAM5.2	Negative
CK20	Negative
Desmin	Negative
Myogenin	Negative
Actin (smooth muscle)	Negative
CD31	Negative
CD34	Negative
CD99	Negative
CD45	Negative
Synatophysin	Negative
Chromogranin	Negative

IHC staining of was negative for melanoma, carcinoma, or other differentiated sarcomas (including those with muscle, neural and vascular differentiation). The bold values are key immunohistochemical stains which point to the diagnosis of UPS.

**Figure 3:**
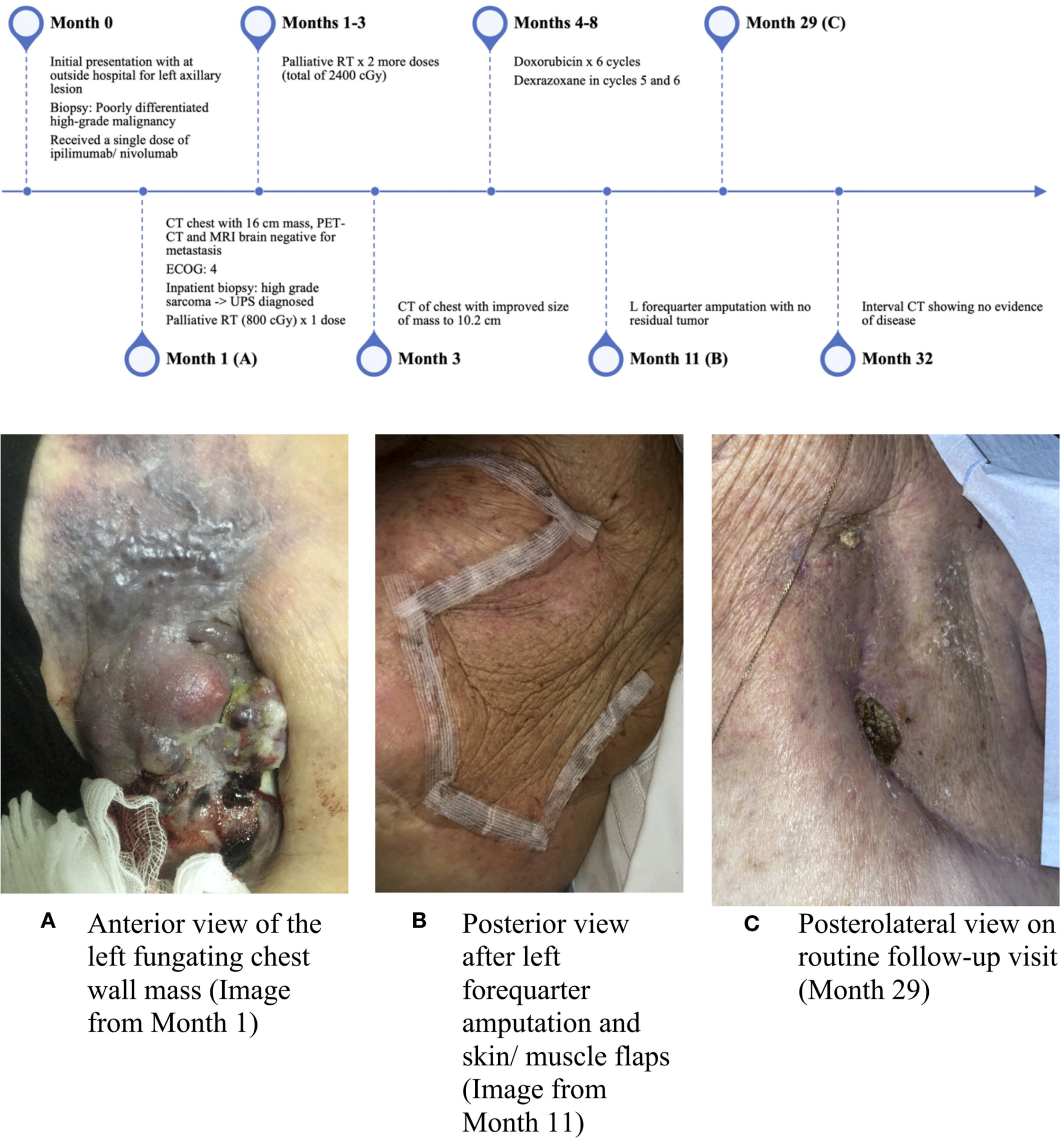
Timeline of events and interval images of the chest wall lesion. **(A)** Anterior view of the left fungating chest wall mass (image from Month 1), **(B)** Posterior view after left forequarter amputation and skin/muscle flaps (image from Month 11), and **(C)** Posterolateral view on routine follow-up visit (Month 29).

At her initial outpatient oncology visit, the patient remained bedbound and completely debilitated, with an ECOG PS of 4. Best supportive care and enrollment in a hospice were recommended; however, due to the significant symptom burden stemming from the left axillary mass, a referral for consideration of additional palliative RT was placed. The patient received two additional doses of palliative RT (for a total of 2,400 cGy). An interval CT of the chest demonstrated a significantly decreased size of the chest wall lesion from 17 cm to 10.2 cm (**Figure 2B**). There was a continued mass effect with displacement of the left subclavian and axillary arteries along with occlusion of the left axillary vein. Given a significant reduction in tumor size and dramatic improvement in PS after three doses of palliative RT, the patient underwent treatment with doxorubicin (75 mg/m^2^ every 21 days) with neoadjuvant intent, which was well tolerated. Dexrazoxane was added in cycles 5 and 6 for cardioprotection. CT of the chest after completion of six cycles of doxorubicin showed similar size of the mass with continued encasement and occlusion of the axillary vein and a stable, indeterminate pulmonary nodule. A moderate left-sided pleural effusion was noted, which resolved after thoracentesis. The cytology was negative for malignant cells, and the pleural effusion was attributed to lymphovascular obstruction from the axillary mass. Given the improvement in tumor size and improved PS, the patient underwent a left forequarter amputation. Pathology demonstrated no residual viable tumor. Necrosis and inflammation were present in the tumor bed, and TILs were estimated at 1%. After over two years of surveillance at the time of this writing, the patient has no evidence of disease (**Figures 2C**, **3**).

## 3 Discussion

Undifferentiated pleomorphic sarcoma is an aggressive malignancy with high metastatic potential. Successful surgical resection is associated with a 5-year disease-free survival (DFS) rate of 54.3%, but outcomes for patients unable to undergo surgery are poor (5). The UPS of the chest wall is often difficult to resect due to the anatomy and surrounding neurovascular structures (6). Pathologic complete response (pCR) is uncommon in STSs after radiation with or without chemotherapy, with pCR rates ranging from 8% to 23% (7, 8). We report a patient with locally advanced UPS of the chest wall who initially received ICI therapy with ipilimumab and nivolumab after being misdiagnosed with cutaneous melanoma at an outside institution. The patient demonstrated striking improvement following palliative-intent RT, which subsequently enabled her to receive neoadjuvant doxorubicin and definitive surgical resection. No viable tumors were seen in the surgical specimen.

The patient’s striking response raises the question as to whether treatment with ipilimumab and nivolumab before RT and chemotherapy “primed” the tumor and tumor microenvironment (TME) for favorable outcomes. Studies have demonstrated that patients with UPS express higher levels of PD-L1 (in about 30%–50% of cases) and PD-1 and increase immune T-cell infiltration (9–11). Recent studies have demonstrated dynamic changes occurring in the TME of UPS tumor cells in response to treatment with RT. In a retrospective study involving 17 patients with UPS involving the trunk or the extremities, Keung et al. demonstrated that tumor cells that did not previously exhibit PD-L1 expression developed PD-L1 expression with increased immune infiltrates following RT (12). The ability of chemotherapy or ICIs to alter the TME of UPS is yet to be validated, but further studies may elucidate the clinical significance of alterations in the TME in response to various treatments.

Currently, the role of targeted therapies such as ICIs is a topic of great interest in UPS. Several small, non-comparative studies have demonstrated the activity of ICIs in advanced UPS. For example, the SARC028 trial reported a 40% overall response rate (ORR) with pembrolizumab in advanced/unresectable UPS (13). In Alliance A091401, a phase II randomized non-comparative trial evaluating nivolumab and nivolumab plus ipilimumab in advanced STSs, confirmed responses were seen in 3/11 (27%) of patients with UPS (14). Checkpoint inhibitors have also demonstrated promising activity in the neoadjuvant setting. In NCT03307616 evaluating 25 patients with resectable STSs (nine patients with UPS) treated with RT plus ICI (either nivolumab alone or ipilimumab plus nivolumab), 95% of patients demonstrated pathologic response; at two-year follow-up, median progression-free survival (PFS) and OS were not reached in UPS patients (15, 16). Pathologic response and radiographic response were poorly correlated, which matches our experience (residual 10 cm mass on imaging, but no viable disease in the surgical specimen). These encouraging results from ICI trials provide a plausible rationale for the exceptional response seen in our patient.

Pathologic complete response was associated with favorable overall survival in a combined analysis of the Radiation Therapy Oncology Group (RTOG) trials 0630 and 9514, which evaluated survival outcomes in STSs for patients receiving preoperative RT or chemoradiotherapy (7). These trials, which included 42 patients with UPS (out of 143), demonstrated 100% 5-year overall survival (OS) in patients who achieved pCR, defined as 0% tumor viability. Any tumor viability greater than 0% was associated with worse OS in both trials (OS of 76.5% and 56.4% in trials 9514 and 0630, respectively) (7). These findings suggest that pCR after chemoradiotherapy or RT alone may be considered a prognostic biomarker for better clinical outcomes.

The relationship between initial ICI therapy and our patient’s excellent response to neoadjuvant radiation and chemotherapy is intriguing. However, we acknowledge the limitations of a single case study, and it is not possible to know whether the patient would have had a similar outcome had ICI therapy been omitted. The randomized SU2C-SARC032 trial aims to assess whether the addition of perioperative pembrolizumab immunotherapy to standard radiation and surgical resection improves DFS in patients with UPS and dedifferentiated/pleomorphic liposarcoma (NCT03092323) (17). The study is currently recruiting, and we eagerly anticipate its results.

## 4 Conclusion

Undifferentiated pleomorphic sarcoma is a challenging malignancy, and outcomes for patients who are not surgical candidates are poor. We report an exceptional response to palliative-intent RT in a patient who had received nivolumab and ipilimumab immunotherapy for a misdiagnosis of melanoma. Despite an initial ECOG PS of 4, she was able to undergo neoadjuvant chemotherapy and ultimately surgical resection. The role that the ICI treatment may have played in her care is intriguing, and early studies have shown evidence of its clinical activity in UPS. Randomized studies evaluating the role of checkpoint inhibitors in the neoadjuvant setting of UPS are ongoing.

## Data availability statement

The original contributions presented in the study are included in the article/supplementary material. Further inquiries can be directed to the corresponding author.

## Ethics statement

Written informed consent was obtained from the participant/patient(s) for the publication of this case report.

## Author contributions

WJ contributed to the original draft preparation and writing. JM, BP, and BJ contributed to the review and editing of the manuscript. LD contributed to the review and editing of the manuscript and provided expert details for the revised manuscript. JB conceptualized the manuscript and supervised the writing and revision of the manuscript. All authors contributed to the article and approved the submitted version.

## References

[1] Siegel RL Miller KD Jemal A . Cancer statistics, 2018. CA Cancer J Clin (2018) 68(1):7–30. doi: 10.3322/caac.21442 29313949

[2] Robles-Tenorio A Solis-Ledesma G . Undifferentiated pleomorphic sarcoma (2022). StatPearls Publishing. Available at: https://www.ncbi.nlm.nih.gov/books/NBK570612/ (Accessed November 29, 2022). 34033374

[3] Clemente O Ottaiano A Di Lorenzo G Bracigliano A Lamia S Cannella L . Is immunotherapy in the future of therapeutic management of sarcomas? J Transl Med (2021) 19(1):173. doi: 10.1186/s12967-021-02829-y 33902630 PMC8077947

[4] Zheng B Zhang S Cai W Wang J Wang T Tang N . Identification of novel fusion transcripts in undifferentiated pleomorphic sarcomas by transcriptome sequencing. Cancer Genomics Proteomics (2019) 16(5):399–408. doi: 10.21873/cgp.20144 31467233 PMC6727074

[5] Lee K Song JS Kim JE Kim W Song SY Lee MH . The clinical outcomes of undifferentiated pleomorphic sarcoma (UPS): a single-centre experience of two decades with the assessment of PD-L1 expressions. Eur J Surg Oncol (2020) 46(7):1287–93. doi: 10.1016/j.ejso.2020.02.029 32127249

[6] Kocaman G Yenigün MB Kaya B Özden NS Özdemir A Kocak ME . A rare giant sarcoma of the chest wall: undifferentiated pleomorphic sarcoma. Turk Gogus Kalp Damar Cerrahisi Derg (2021) 29(4):552–5. doi: 10.5606/tgkdc.dergisi.2021.20061 PMC8762898 35096456

[7] Wang D Harris J Kraybill WG Eisenberg B Kirsch DG Ettinger DS . Pathologic complete response and clinical outcomes in patients with localized soft tissue sarcoma treated with neoadjuvant chemoradiotherapy or radiotherapy: the NRG/RTOG 9514 and 0630 nonrandomized clinical trials. JAMA Oncol (2023) 9(5):646–55. doi: 10.1001/jamaoncol.2023.0042 PMC10064284 36995690

[8] Bonvalot S Wunder J Gronchi A Broto JM Turcotte R Rastrelli M . Complete pathological response to neoadjuvant treatment is associated with better survival outcomes in patients with soft tissue sarcoma: results of a retrospective multicenter study. Eur J Surg Oncol (2021) 47(8):2166–72. doi: 10.1016/j.ejso.2021.02.024 33676792

[9] Pollack SM He Q Yearley JH Emerson R Vignali M Zhang Y . T-Cell infiltration and clonality correlate with programmed cell death protein 1 and programmed death-ligand 1 expression in patients with soft tissue sarcomas. Cancer (2017) 123(17):3291–304. doi: 10.1002/cncr.30726 PMC5568958 28463396

[10] Movva S Wen W Chen W Millis SZ Gatalica Z Reddy S . Multi-platform profiling of over 2000 sarcomas: identification of biomarkers and novel therapeutic targets. Oncotarget (2015) 6(14):12234–47. doi: 10.18632/oncotarget.3498 PMC4494935 25906748

[11] Roulleaux Dugage M Nassif EF Italiano A Bahleda R . Improving immunotherapy efficacy in soft-tissue sarcomas: a biomarker driven and histotype tailored review. Front Immunol (2021) 12:775761. doi: 10.3389/fimmu.2021.775761 34925348 PMC8678134

[12] Keung EZ Tsai JW Ali AM Cormier JN Bishop AJ Guadagnolo BA . Analysis of the immune infiltrate in undifferentiated pleomorphic sarcoma of the extremity and trunk in response to radiotherapy: rationale for combination neoadjuvant immune checkpoint inhibition and radiotherapy. Oncoimmunology (2017) 7(2):e1385689. doi: 10.1080/2162402X.2017.1385689 29308306 PMC5749668

[13] Tawbi HA Burgess M Bolejack V Van Tine BA Schuetze SM Hu J . Pembrolizumab in advanced soft-tissue sarcoma and bone sarcoma (SARC028): a multicentre, two-cohort, single-arm, open-label, phase 2 trial. Lancet Oncol (2017) 18(11):1493–501. doi: 10.1016/S1470-2045(17)30624-1 PMC7939029 28988646

[14] D'Angelo SP Mahoney MR Van Tine BA Atkins J Milhem MM Jahagirdar BN . Nivolumab with or without ipilimumab treatment for metastatic sarcoma (Alliance A091401): two open-label, non-comparative, randomised, phase 2 trials. Lancet Oncol (2018) 19(3):416–26. doi: 10.1016/S1470-2045(18)30006-8 PMC6126546 29370992

[15] Roland CL Keung EZY Lazar AJ Torres KE Wang WL Guadagnolo A . Preliminary results of a phase II study of neoadjuvant checkpoint blockade for surgically resectable undifferentiated pleomorphic sarcoma (UPS) and dedifferentiated liposarcoma (DDLPS). JCO (2020) 38(15_suppl):11505–5. doi: 10.1200/JCO.2020.38.15_suppl.11505

[16] Keung EZY Nassif EF Lin HY Lazar AJ Torres KE Wang WL . Randomized phase II study of neoadjuvant checkpoint blockade for surgically resectable undifferentiated pleomorphic sarcoma (UPS) and dedifferentiated liposarcoma (DDLPS): survival results after 2 years of follow-up and intratumoral b-cell receptor (BCR) correlates. J Clin Oncol (2022) 40(17_suppl):LBA11501–LBA11501. doi: 10.1200/JCO.2022.40.17_suppl.LBA11501

[17] Mowery YM Ballman KV Riedel RF Brigman BE Attia S Meyer CF . SU2C-SARC032: a phase II randomized controlled trial of neoadjuvant pembrolizumab with radiotherapy and adjuvant pembrolizumab for high-risk soft tissue sarcoma. J Clin Oncol (2018) 36(15_suppl):TPS11588–TPS11588. doi: 10.1200/JCO.2018.36.15_suppl.TPS11588

